# Supplementary material for: Trend Distribution of Violent Injuries in Taiwan from 2000 to 2015
Source: Int J Environ Res Public Health. 2022 Jun 27;19(13):7874. doi: 10.3390/ijerph19137874 (PMC9265989; doi:10.3390/ijerph19137874)
Supplement: Supplementary file 1 [file ijerph-19-07874-s001.zip › ijerph-1696064-supplementary.pdf]

Table S1. Injured site and injury severity (RISS) of children and adolescents injured by violence

Injury status (location, severity)		*0-5.9 years old*	*(n =44)*	*6-11.9 years old*	*(n =173)*	*12-17.9 years old*	*(n =1375)*	*p*
		n	*%*	*n*	*%*	*n*	*%*	
Injured area‡	*Head/Neck*	*6*	***13.6***	*19*	***11.0***	*256*	***18.6***	*0.0357**
	Face	*0*	*0.0*	*3*	*1.7*	*57*	*4.1*	*0.1203*
	Thorax	*0*	*0.0*	*7*	*4.0*	*56*	*4.1*	*0.7844*
	Abdomen	*3*	*6.8*	*7*	*4.0*	*52*	*3.8*	*0.5880*
	Extremity	*3*	*6.8*	*9*	*5.2*	*146*	*10.6*	*0.0643*
	Appearance Soft Tissue (External)	*12*	***27.3***	*59*	***34.1***	*391*	***28.4***	*0.2918*
Injury severity	*(RISS)*							
	No trauma (ISS=0)	*20*	*45.5*	*83*	*48.0*	*739*	***53.7***	*0.1309* [a]
	Minor trauma (ISS ≦ 8)	*20*	*45.5*	*81*	*46.8*	*543*	*39.5*	
	Moderate trauma (ISS 9-15)	*4*	*9.1*	*9*	*5.2*	*93*	*6.8*	
	Serious trauma (≧ 16)	*0*	*0.0*	*0*	*0.0*	*0*	*0.0*	

*by chi-square test, *: $p < 0.05$, a: Fisher exact test. Soft tissue refers to the human body's muscles, fascia, tendons, ligaments, tendon sheaths, bursae, blood vessels, nerves and other tissues. ‡ Check.*

Table S2. Injured location, injury severity (RISS) of adult victims of violence

Injury status (location, severity)		*18-23.9 years old (n =1509)*		*24-44.9 years old (n =4661)*		*45-64.9 years old (n =2556)*		*p*
		n	*%*	*n*	*%*	*n*	*%*	
Injured area‡	*Head/Neck*	*405*	*26.8*	*1227*	*26.3*	*631*	*24.7*	*0.2140*
	Face	*66*	*4.4*	*208*	*4.5*	*107*	*4.2*	*0.8598*
	Thorax	*125*	*8.3*	*449*	*9.6*	*305*	*11.9*	*0.0003***
	Abdomen	*77*	*5.1*	*307*	*6.6*	*125*	*4.9*	*0.0055***
	Extremity	*295*	*19.5*	*836*	*17.9*	*467*	*18.3*	*0.3701*
	Appearance Soft Tissue (External)	*711*	*47.1*	*2199*	*47.2*	*1104*	*43.2*	*0.0032***
Injury severity	*(RISS)*							
	No trauma (ISS=0)	*450*	*29.8*	*1265*	*27.1*	*736*	*28.8*	*0.0016***
	Minor trauma (ISS ≦ 8)	*883*	*58.5*	*2836*	*60.8*	*1494*	*58.5*	
	Moderate trauma (ISS 9-15)	*140*	*9.3*	*398*	*8.5*	*207*	*8.1*	

	Serious trauma (≧ 16)	*36*	*2.4*	*162*	*3.5*	*119*	*4.7*	

*by chi-square test, **: $p < 0.01$. Soft tissue refers to the human body's muscles, fascia, tendons, ligaments, tendon sheaths, bursae, blood vessels, nerves and other tissues. ‡ Check.*

Table S3. Injured location and injury severity (RISS) of the elderly victims of violence

Injury status (location, severity)		*65-74.9 years old (n =432)*		*75-84.9 years old (n =265)*		*85+ (n =62)*		*p*
		n	*%*	*n*	*%*	*n*	*%*	
Injured area‡	*Head/Neck*	*85*	*19.7*	*44*	*16.6*	*10*	*16.1*	*0.5348*
	Face	*18*	*4.2*	*6*	*2.3*	*0*	*0.0*	*0.1258*
	Thorax	*45*	*10.4*	*24*	*9.1*	*3*	*4.8*	*0.3584*
	Abdomen	*23*	*5.3*	*9*	*3.4*	*0*	*0.0*	*0.1063*
	Extremity	*84*	*19.4*	*41*	*15.5*	*11*	*17.7*	*0.4140*
	Appearance Soft Tissue (External)	*169*	*39.1*	*94*	*35.5*	*18*	*29.0*	*0.2483*
Injury severity	*(RISS)*							
	No trauma (ISS=0)	*142*	*32.9*	*109*	*41.1*	*32*	*51.6*	*0.0134**
	Minor trauma (ISS ≦ 8)	*230*	*53.2*	*123*	*46.4*	*19*	*30.6*	
	Moderate trauma (ISS 9-15)	*41*	*9.5*	*18*	*6.8*	*6*	*9.7*	
	Serious trauma (≧ 16)	*19*	*4.4*	*15*	*5.7*	*5*	*8.1*	

*by chi-square test, *: $p < 0.05$. Soft tissue refers to the human body's muscles, fascia, tendons, ligaments, tendon sheaths, bursae, blood vessels, nerves and other tissues. ‡ Check.*
